# Clinical and Virological Response to Convalescent Plasma in a Chronic Lymphocytic Leukemia Patient with COVID-19 Pneumonia

**DOI:** 10.3390/life12071098

**Published:** 2022-07-21

**Authors:** Giovanni Belcari, Alberto Conti, Alessandro Mazzoni, Maria Lanza, Paola Mazzetti, Daniele Focosi

**Affiliations:** 1 Division of Emergency Medicine, ASL Toscana Nord-Ovest, 56124 Pisa, Italy; giovannibelcari@hotmail.com; 2 Department of Emergency Medicine, ASL Toscana Nord-Ovest, 56124 Pisa, Italy; alberto.conti@uslnordovest.toscana.it; 3 Division of Transfusion Medicine, Pisa University Hospital, 56124 Pisa, Italy; a.mazzoni@ao-pisa.toscana.it; 4 North-Western Tuscany Blood Bank, Pisa University Hospital, 56124 Pisa, Italy; m.lanza@ao-pisa.toscana.it; 5 Division of Virology, Pisa University Hospital, 56124 Pisa, Italy; p.mazzetti@ao-pisa.toscana.it

**Keywords:** COVID-19, SARS-CoV-2, chronic lymphocytic leukemia, venetoclax, convalescent plasma, immunosuppression

## Abstract

The burden of COVID-19 remains unchanged for immunocompromised patients who do not respond to vaccines. Unfortunately, Omicron sublineages are resistant to monoclonal antibodies authorized in Europe so far, and small chemical antivirals have contraindications and toxicities that have not been studied in these patients. We report here the successful treatment of COVID-19 pneumonia lasting for 4 months after the transfusion of COVID-19 convalescent plasma (CCP) in a patient with severe immunosuppression due to both chronic lymphocytic leukemia and venetoclax treatment. The patient achieved a complete clinical, radiological and virological response after six transfusions (600 mL each) of high-titer CCP collected from triple-vaccinated and convalescent donors. This dramatic case adds to the mounting evidence of CCP efficacy in immunocompromised patients, provided that high-titer and large volumes are infused.

The COVID-19 pandemic has caused 554 million cases and 6.35 million deaths worldwide, as of 8 July 2022. While most immunocompetent subjects who have been triple-vaccinated do not progress to severe disease after SARS-CoV-2 infection, immunocompromised patients, and especially the ones with B-cell depletion, do not respond to vaccines, and remain hence at increased risk of complications [1]. Such frail patients often have contraindications to or cannot tolerate the toxicity of small chemical antivirals, for which safety and efficacy issues still persist. Additionally, the advent of the Omicron sublineages of SARS-CoV-2 has caused a loss of efficacy for all therapeutic anti-SARS-CoV-2 Spike monoclonal antibodies (mAbs) authorized so far in Europe [2,3]. In this scenario, there is an urgent need for novel therapeutic agents. COVID-19 convalescent plasma (CCP) has been initially investigated as a treatment for COVID-19 inpatients [4], but findings from randomized controlled trials (RCT) have consistently shown definite efficacy only in outpatients treated within 5 days from onset of symptoms [5,6,7]. This is in line with indications for the usage of any other antiviral, including small-chemical and anti-Spike mAbs. On the basis of encouraging case reports and large case series [8,9], the FDA re-authorized CCP for immunosuppressed patients in January 2022 [10].

We report here the case of a 62-year-old male patient with chronic lymphocytic leukemia. He had been treated with rituximab plus bendamustine in 2015 and in 2019, with partial responses. In January 2021, the patient experienced a second relapse (showing de novo mutation of TP53 and 13q14.2 microdeletion) and received chlorambucil debulking before starting rituximab plus venetoclax (400 mg) in March 2021. The last (third) rituximab infusion was in September 2021. In October 2021 the patient received his third dose of BNT162b2, but anti-Spike antibodies remained undetectable a few months later. On December 31, he tested positive for SARS-CoV-2 RNA via a nasopharyngeal swab (NPS). He was not admitted at hospital, was left at home under low-molecular-weight heparin, and received no small-chemical antiviral or anti-Spike mAb; while at home, despite azithromycin and ciprofloxacin courses, he became febrile and required low-flow oxygen support with nasal cannulas every day for 3 months. On 20 April 2022, a chest CT scan performed at the emergency room in Portoferraio showed diffuse interstitial pneumonia, and type 1 respiratory failure was seen in his arterial blood gases (ABG: pO2 64 mmHg, pCO2 33 mmHg, pH 7.6). Venetoclax was discontinued on April 24. The viral load on the NPS was very high (Ct = 19) and SARS-CoV-2 was also found in the peripheral blood (Ct = 38). Whole-genome sequencing of the virus showed that the strain belonged to BA.1. While such lineage was still sensitive to sotrovimab, the drug was not available at local hospitals at that time, and there was mounting evidence of treatment-emergent immune escape to sotrovimab in immunocompromised patients [11,12]. Small-chemical antivirals such as molnupiravir and nirmatrelvir/ritonavir were not offered because of poor efficacy [13] and contraindications, respectively; none of the pills had been investigated in immunosuppressed patients or for exposures longer than 5 days, which was likely needed in our patient because of the high initial viral load.

The ethical committee of North-Western Tuscany granted permission to use CCP for this patient on April 24. The patient received six CCP units: the first (600 mL; >5500 BAU WHO/mL) on April 25, the second on May 1 (two 300 mL aliquots from the same donation; anti-authentic live Omicron-neutralizing antibody titer 1:640), the third on May 8 (600 mL; 3296 BAU WHO/mL), the fourth on May 23 (600 mL; >5500 BAU/mL), the fifth on May 30 (1279 BAU WHO/mL), and the sixth on June 4 (600 mL; >5500 BAU/mL). The first and fourth units were from the same donor. All donors were triple-vaccinated and convalescent from different SARS-CoV-2 VOCs. A few hours after the first CCP transfusion, the patient was apyretic (despite the suspension of corticosteroids and paracetamol), and oxygen supplementation was discontinued from April 29. A follow-up chest CT scan on May 9 showed the resolution of pneumonia, despite the persistence of focal fibrosis (Figure 1). Table 1 shows the progressive decrease in viral load and inflammatory serum markers with CCP treatment. Immunophenotyping by flow cytometry on May 22 confirmed a 0% presence of CD19+ B-lymphocytes. The sudden changes could not be explained by immune reconstitution after the discontinuation of venetoclax, and especially the sudden drop in viral load can only be attributed to neutralizing antibodies within the transfused CCP units. 

In a recent RCT on 133 immunosuppressed patients, two 300 mL plasma units (collected from either convalescent or double-vaccinated donors) with nAb titers > 1:80 led to a clinically significant benefit in patients with hematological malignancies, other cancers, or immunosuppression (group 1, group 2, *n* = 71), with a median time to improvement of 10 days for vaccinated plasma, 13 days for CCP, and 32 days for the control [14].

Mounting evidence has shown the superiority of hybrid plasma (i.e., CCP collected from donors who are both convalescent and at least double-vaccinated [15]), and the relevance of dose in CCP treatment [16]. Our patients received six units of plasma collected from donors who had been both infected and triple-vaccinated, and accordingly showed very high anti-Spike antibody levels.

This case primed the startup of a dedicated path within the hospital network of the ASL Toscana Nord-Ovest, starting from CCP collection and resulting in stocking for transfusion within dedicated ambulatories [17].

In conclusion, we have shown here that CCP, a treatment with previously proven benefit in immunocompetent patients when given in the first 5 days from the onset of symptoms (when the patients are more likely to be seronegative), can be a life-saving treatment in immunocompromised patients, especially ones with B-cell depletion. In immunocompromised patients, benefit from CCP is expected at far later timepoints than in immunocompetent patients, likely until they become seronegative, in line with the principles of replacement therapy. While there are no standardized doses and schedules for CCP yet [16], our case suggests that the duration of treatment should be driven by virological response (Ct in PCR).

## Figures and Tables

**Figure 1:**
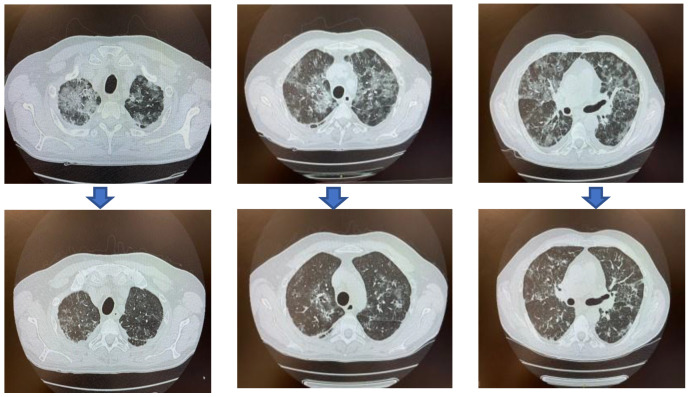
Changes in chest CT scan before (upper row) and after (lower row) CCP treatment.

**Table 1:** Cycle threshold (Ct) values from qualitative PCR serial nasopharyngeal swabs and other laboratory biomarkers alongside CCP treatment. n.a.—not available.

	Jan 30	April 25	April 30	May 5	May 9	May 14	May 20	May 26	May 31	June 4	Jun 12
E-gene probe (Ct)		19.6	24.8	27	26.8	27.7	37.0	36.8	neg	neg	neg
N2-gene probe (Ct)		21.6	27.4	29.3	29.7	30.6	39.0	38.9	42.5	42	neg
lymphocytes/mcl	600	100	300	n.a.	600	n.a.	1400	n.a.	n.a.	n.a.	n.a.
high-sensitivity C-reactive protein (mg/dL)	4.31	15.78	4.49	n.a.	0.58	n.a.	n.a.	n.a.	n.a.	n.a.	n.a.
D-dimer (ng/mL)	694	2777	1366	n.a.	1247 ng/mL	n.a.	n.a.	n.a.	n.a.	n.a.	n.a.

## Data Availability

Data are available via corresponding author.

## Abbreviation

CCP	COVID-19 convalescent plasma
